# Canola Meal: A Sustainable Protein Source for Poultry Diets

**DOI:** 10.3390/ani15243609

**Published:** 2025-12-15

**Authors:** Thi Hiep Dao, Amy F. Moss

**Affiliations:** 1 School of Environmental and Rural Science, Faculty of Science, Agriculture, Business and Law, University of New England, Armidale, NSW 2351, Australia; tdao2@une.edu.au; 2 Faculty of Animal Science, Vietnam National University of Agriculture, Ngo Xuan Quang Street, Gia Lam Commune, Hanoi 100000, Vietnam; 3 Poultry Hub Australia, University of New England, Armidale, NSW 2350, Australia

**Keywords:** canola meal, rapeseed, layer hen, broiler chicken, sustainability, nutrition, economic, environment

## Abstract

Soybean meal has traditionally dominated poultry diets as the protein source. However, its widespread use raises concerns regarding economic costs, environmental impact and social sustainability. As a result, there is growing interest in alternative protein sources, such as canola meal, which may reduce feed costs while sustaining productivity. This review evaluates the potential of canola meal as a sustainable protein source in modern poultry production systems, focusing on nutritional, economic and environmental advantages, as well as the potential implications of canola meal inclusion in reduced-protein diets. Evidence from scientific studies indicates that canola meal’s nutritional profile supports bird growth and production, although higher fiber content and anti-nutritional compounds reduce metabolizable energy, making it more suitable for laying hens than broiler chickens. Processing techniques, enzyme supplementation, fermentation, and modern cultivars have improved both nutritional value and practical utility of canola meal. Performance outcomes differ by species. Broilers exhibit variable growth at high inclusion levels, whereas laying hens are estimated to tolerate up to 20% without affecting laying performance or egg quality; however, data is severely lacking, particularly under the context of modern reduced-protein diets. Economically, canola meal is cost-competitive with soybean meal. From an environmental perspective, substituting imported soybean meal with local canola reduces greenhouse gas emissions, enhances resource efficiency, and supports pollinators. Nevertheless, trade-offs exist, including increased land use, variable digestibility, and potential eutrophication. Incorporating canola meal into reduced-protein diets offers both economic and ecological benefits, though effectiveness depends on the extent of protein reduction and the precision of amino acid formulation. Overall, canola meal offers a sustainable, economically viable, and environmentally responsible protein source for modern poultry production, provided that inclusion levels are adjusted to species-specific requirements and regional conditions.

## Simple Summary

Although soybean meal continues to underpin global poultry nutrition, reliance on it carries mounting environmental and economic risks. Human dietary shifts toward plant-based foods may also intensify land-use competition and amplify sustainability challenges, even in regions with fixed arable land boundaries such as the USA. Together, these pressures highlight the importance of developing alternative protein sources for poultry production. Canola meal offers one such alternative protein source. This review assesses the role of canola meal as an alternative protein source in poultry nutrition, highlighting its nutritional, economic, and environmental attributes, and examining its potential within reduced-protein feeding strategies. Evidence from the literature indicates that canola meal constitutes a viable and adaptable alternative protein source in poultry diets, providing nutritional, economic, and environmental benefits when applied appropriately.

## 1. Introduction

Concerns about resource efficiency and the environmental footprint of livestock production continue to escalate. A major challenge lies in the competition between crops grown for human food and those destined for animal feed, particularly regarding land and energy use [1]. This issue has gained urgency in light of the planet’s limited ecological capacity and the instability of global systems. Geopolitical conflicts, market disruptions, social inequalities, rising inflation and energy shortages further threaten feed supply chains and global food security [2]. Within this context, soybean meal has drawn significant scrutiny [3]. It remains the dominant protein supplement in poultry diets, yet its sustainability has become contested. The production of soybean meal is associated with a wide range of negative impacts, including deforestation, soil degradation, greenhouse gas emissions, acidification, eutrophication, and biodiversity loss [4,5]. Although soybean meal continues to underpin global poultry nutrition, reliance on it carries mounting environmental and economic risks [6,7]. Human dietary shifts toward plant-based foods may also intensify land-use competition and amplify sustainability challenges, even in regions with fixed arable land boundaries such as the USA [8]. Together, these pressures highlight the importance of developing alternative protein sources for poultry production.

Canola meal, produced as a co-product of oil extraction, offers one such alternative protein source [9]. Australia is a major contributor to the global canola trade, accounting for 15–20% of exports [10]. Earlier work showed that nearly half of the canola meal produced locally was incorporated into poultry feed [11]. More recently, Swick and Wu [12] reported that in 2014, Australia produced approximately 3.42 million tonnes of canola seed, yet the corresponding output of canola meal was comparatively small, at about 490,000 tonnes [12]. Historically, low available energy and the presence of anti-nutritional compounds limited wider adoption of canola meal in poultry diets [13]. However, advances in plant breeding and processing have reduced erucic acid, glucosinolates, and fiber levels, improving nutritional value and feeding potential of canola meal [14,15,16,17]. This progress creates new opportunities for greater utilization of canola meal in Australian poultry industry and other parts of the world where canola can be produced locally and less reliance on imported soybean meal [9]. Economic aspects further strengthen its relevance. Importing soybean meal involves high transport costs and exposes producers to volatile fuel markets and trade disruptions [18]. By contrast, canola meal processed locally can reduce costs and strengthen supply stability [16]. Its use also supports circular economy principles by making full use of oil-processing co-products, thereby linking poultry feeding to regional agricultural systems.

Interest in reduced-crude-protein (CP) diets for poultry has grown considerably in recent years, reflecting a dual emphasis on sustaining productive performance while enhancing the environmental sustainability of poultry production systems [19,20]. Economic factors are also shaping this transition. As the cost of crystalline amino acids continues to decline, and as soybean meal prices trend upward, the adoption of reduced-CP feeding strategies is expected to become increasingly feasible [21]. Nutritionally, canola meal provides relatively high levels of methionine and cysteine, amino acids often limiting in cereal-based feeds [22,23]. This is especially relevant in Australia and the regions, where wheat is the main feed grain and protein reduction efforts are frequently constrained by its amino acid profile [24]. By complementing wheat, canola meal contributes to improved amino acid balance and supports the adoption of reduced-protein feeding systems. In doing so, it offers scope to decrease feed costs and mitigate environmental impacts without sacrificing productivity. This review assesses the role of canola meal as an alternative protein source in poultry nutrition, highlighting its nutritional, economic, and environmental attributes, and examining its potential within reduced-protein feeding strategies.

## 2. Canola: Past and Present Use

Canola represents a cultivar group derived from rapeseed (*Brassica* spp.), a member of the Brassicaceae family that also includes several agronomically important crops such as mustard, cabbage and kale [25]. The cultivation of rapeseed dates back more than three thousand years in India and approximately two thousand years in regions of China and Japan. Broader industrial acceptance emerged during the era of steam power, when rapeseed oil gained importance as a reliable lubricant. The introduction of rapeseed into Canada occurred in the late 1930s and early 1940s, primarily to diversify cropping systems within the Prairie Provinces [25,26,27]. Traditional rapeseed varieties, however, were constrained in their use as food or feed because of high levels of erucic acid and glucosinolates. Hydrolysis of glucosinolates by the enzyme myrosinase generates metabolites with goitrogenic effects, which interfere with iodine utilization, impair thyroid function, and reduce animal performance [28]. To overcome these challenges, Canadian plant breeders initiated efforts to develop cultivars with reduced concentrations of these anti-nutritional compounds. Pioneering work by Dr. Baldour R. Stefansson at the University of Manitoba led to the release of low-erucic acid rapeseed cultivars, earning him recognition as the “father of canola.” By 1968, early cultivars such as Tanka, Target and Turret were released, followed by the first double-low cultivar—Tower—in 1974, which combined low glucosinolate and low erucic acid traits [25]. The name “canola” was introduced in 1979 as a contraction of “Canada” and “ola” (denoting “oil low acid”) and was adopted to distinguish these improved cultivars from conventional rapeseed [29]. According to the Canola Council of Canada [29], canola is defined as *Brassica napus*, *B. rapa*, or *B. juncea* cultivars that produce oil containing less than 2% erucic acid and meal containing less than 30 µmol/g of specified aliphatic glucosinolates, calculated on an oil-free, air-dry basis. These varieties are also known internationally as “double-low” or “double zero” rapeseed [29].

Canola is currently the third most significant oilseed crop worldwide, and its production has expanded markedly across major producing regions over recent decades [30]. Since 1993, output has increased approximately twofold in China (reaching 14.5 million tonnes), threefold in Canada (17.9 million tonnes), and fourfold in the European Union (20.9 million tonnes). During the same period, growth in Australia has been particularly striking, with production rising more than tenfold from 0.3 to 4.0 million tonnes per year, making canola the nation’s third most valuable grain crop after wheat and barley [31,32]. Recently, Australia has emerged as one of the world’s leading producers, with harvests reaching 8.3 million tonnes in 2023 [32,33]. The wide availability of the crop is one of the reasons it is now a regular feedstuff included to Australian poultry diets, discussed later in the review. The nutritional profile of Australian canola seed has been examined in detail by Toghyani et al. [34], who analyzed 11 seed samples from different regions and reported average values of 21.5% CP, 7016 kcal/kg gross energy, and 42.9% crude fat. Processing canola seed generally yields about 42% oil, widely consumed as edible vegetable oil, and approximately 58% meal, which is marketed as a high-protein feedstuff for a variety of livestock species including poultry, pigs and cattle [26,35]. The competitiveness of canola meal relative to soybean meal largely depends on its nutrient composition and market price [36]. In the Australian poultry sector, both canola seed and canola meal are regularly incorporated into feed formulations [9].

## 3. Nutritional Characteristics

### 3.1. Crude Protein Level and Amino Acid Digestibility

Canola meal is produced as a co-product during oil extraction from canola seed, which typically contains approximately 40% oil and 17–26% CP [37]. The meal is widely used in animal diets and is increasingly valued in discussions of circular bioeconomy, given its dual role in nutrient provision and resource efficiency. The amino acid composition of canola meal is relatively balanced, supporting its use in poultry diets [13]. Nevertheless, the higher fiber content compared with soybean meal lowers metabolizable energy level in canola meal, which makes it more appropriate for laying hens than for broilers [38]. Dietary fiber is traditionally defined as the combined fraction of non-starch polysaccharides and lignin [9]. In canola meal, non-starch polysaccharides constitute the major portion of this fraction, contributing roughly 53% to 80% of total dietary fiber, whereas only a small proportion (around 10% or less) is water-soluble [39]. These polysaccharides are minimally digested by poultry, with apparent total tract digestibility ranging from 2.1% to 8.6% [40]. Lignin, a structurally complex group of polyphenolic polymers, is likewise non-digestible in poultry [9]. High fiber content can interfere with digestive function and nutrient use [41]. In poultry, fiber-rich diets stimulate goblet cell activity, increasing mucin secretion [42]. Excess mucin forms a thicker unstirred layer at the mucosal surface, which slows the movement of digestive enzymes and substrates and consequently limits nutrient hydrolysis and absorption [43]. Moreover, the high fiber content in canola meal may increase digesta passage, leaving less time for enzymatic breakdown. This faster transit can also increase endogenous nitrogen losses through abrasive effects or through interactions that bind endogenous proteins, ultimately reducing nutrient utilization [13,44].

Canadian Canola Council guidelines specify a minimum CP level of 36% at 12% moisture; the actual CP level of canola meal frequently ranges between 37% and 40% [45]. This variation is influenced by agronomic practices, environmental conditions, harvest timing and processing methods [45]. The variation in the nutrient content of Australian feed ingredients across several databases was explored in Moss [46] and clearly demonstrates the variation in amino acids within canola meal. While the amino acid profile of canola meal generally aligns with poultry requirements, concentrations of lysine and arginine are typically lower than those found in soybean meal [13]. In contrast, canola meal is comparatively rich in sulfur-containing amino acids such as methionine and cysteine, which are often limiting in cereal-based diets [22,23]. This characteristic creates opportunities for complementarity when canola meal is incorporated alongside grain ingredients. Table 1 provides a comparative summary of the nutrient profiles of canola meal and soybean meal [46]. From a formulation perspective, recognizing the strengths and limitations of canola meal’s amino acid profile allows for more efficient protein utilization and can lessen reliance on crystalline amino acid supplementation. Such strategies not only improve the nutritional value of poultry diets but also support cost-effectiveness within intensive production systems.

Research indicates that the digestibility of amino acids in canola meal is generally lower and more variable compared with soybean meal [13]. In laying hens, standardized ileal digestibility values of essential amino acids in canola meal have been reported to range between 78% and 84% [38]. Of which, arginine (88%) and methionine (87%) showed the greatest digestibility, whereas the digestibility values of lysine (74%) and threonine (75%) were lowest. Considerable variability was noted for threonine (68–79%) and lysine (68–78%) digestibility across different canola meal samples [38]. Comparable findings were observed in broiler chickens, where pre-caecal amino acid digestibility in 14 Australian canola meal samples varied from 68% to 83% [47]. Lysine digestibility again showed the widest variation, with values spanning 64% to 84% [47]. Processing conditions and cultivar differences contribute strongly to this variability. Excess heat during oilseed meal production can reduce both amino acid levels and their availability [48]. For example, solvent extraction has been shown to reduce lysine digestibility in canola meal by around 5% [49]. Similarly, desolventization and toasting treatments have been linked to declines in apparent lysine digestibility in canola meal from 87% to 79% [22]. The temperatures used during this stage, usually between 100 and 110 °C, contribute to this decline [22]. Heat can also trigger chemical changes. During processing or extended storage, heat-sensitive amino acids such as lysine may be converted into biologically unavailable derivatives [50]. In contrast to these detrimental effects, some processing techniques may enhance nutrient availability. Ahmed et al. [51] showed that extrusion of canola meal increased ileal amino acid digestibility in broiler chickens. Specifically, extrusion increased the mean apparent ileal digestibility rate across 18 amino acids by 11.3% (77.6% vs. 69.7%), with threonine showing the greatest improvement at 24.1% (70.6% vs. 56.9%) [51]. Such observations highlight the importance of processing methods in evaluating the feeding value of canola meal. Digestibility also differs among poultry species. Although overall amino acid digestibility values of canola meal were not markedly different between broilers and laying hens, laying hens displayed higher digestibility for methionine, tryptophan, and histidine [52]. Ducks, on the other hand, showed greater endogenous amino acid losses but improved ileal digestibility of cysteine and proline compared with broilers [53]. These species-specific responses reflect underlying differences in gastrointestinal physiology, digesta transit, and enzyme activity, and they point to the need for targeted formulation strategies. Nutrient availability from canola meal can be improved by various interventions. Approaches such as controlled heat application, enzymatic treatments, or supplementation with exogenous enzymes have been reported to enhance amino acid utilization [9]. However, practical implementation requires balancing nutritional gains against economic costs within each production context.

### 3.2. Energy Content

The metabolizable energy (ME) content of canola meal is an important factor in poultry nutrition and directly affects diet formulation and feed cost evaluations. On average, solvent-extracted canola meal provides approximately 2000 kcal/kg, which is around 230 kcal/kg less than non-dehulled soybean meal [54]. Expeller-pressed canola meal generally shows higher ME values than solvent-extracted meal due to its greater residual oil concentration [55]. Genetic improvements, including the development of low-glucosinolate cultivars, have also contributed to more favorable ME values [56]. Dehulling further improves the energy profile of canola meal by reducing fiber levels. Standard canola meal supplies roughly 3346 kcal/kg of digestible energy, whereas dehulled fractions provide about 4063 kcal/kg, reflecting the removal of fibrous hulls that constitute 12–16% of the seed [56]. Processing conditions additionally influence energy availability. Conditioning canola seeds at moderate temperatures (95 °C) has been shown to produce higher ileal digestible energy, apparent metabolizable energy (AME), and nitrogen-corrected AME (AMEn) in broiler chickens compared with both lower (90 °C) and higher (100 °C) temperatures. Similarly, increased screw torque during processing enhanced energy utilization in broilers relative to lower torque settings [57]. Relative to soybean meal, canola meal consistently exhibits lower ME values, largely attributable to its higher fiber content and, to a lesser extent, reduced protein concentration [13]. Fiber in canola meal appears to limit energy digestibility, with studies reporting a clear inverse relationship between fiber fraction and energy availability [58]. One explanation for the lower ME is that high dietary fiber can increase digesta passage. Faster passage reduces the time available for digestion, which in turn limits energy utilization [13]. In addition, glucosinolate levels in canola meal have been reported to correlate negatively with AME level, further contributing to the lower energy value [57]. Nonetheless, energy deficits can be mitigated through precise formulation strategies that balance nutrient density, dietary cost, and bird performance. Variation in energy utilization also occurs across bird ages. Khalil et al. [59] reported that AMEn values for both soybean and canola meals follow a quadratic trend with broiler age: highest in the first week after hatch, declining until week three, and subsequently increasing through week six. These findings emphasize the importance of age-specific formulation to optimize both energy efficiency and the economic value of canola meal in poultry production systems.

### 3.3. Carbohydrate and Crude Fiber Level

Canola meal contains a diverse array of carbohydrate fractions, many of which are structurally complex. The fiber component is particularly significant and includes lignin with associated polyphenols (8%), cellulose (4–6%), and non-cellulosic polysaccharides (13–16%), with the latter largely composed of pectic substances [60,61]. Because of the canola’s small seed size and relatively high oil concentration (42–45%), the residual meal retains high fiber content. Canola meal exhibits higher concentrations of crude fiber, acid detergent fiber, neutral detergent fiber, and total dietary fiber compared with soybean meal. This distinction is largely explained by the greater lignin fraction and its associated phenolic compounds, including tannins [13]. The nutritional implications of this composition have been well documented. Fiber fractions in canola meal are negatively associated with energy digestibility [58], and high fiber levels remain one of the principal constraints limiting its wider use in poultry diets [62,63]. Beyond fiber, other constituents of nutritional relevance include oligosaccharides (2.5%), glycoproteins (approximately 5%, e.g., arabinogalactan-proteins and cell wall proteins), phytate (3.3%), fiber-bound minerals (1%), and gum substances (4%) [64]. Like many plant-derived feed ingredients, the carbohydrate composition is not fixed and varies depending on processing techniques, analytical methods, cultivar genetics, harvest timing and environmental conditions during seed development [56]. Canola seed hulls contribute a large proportion of the fiber fraction, consisting of carbohydrate polymers along with proteins and non-starch polysaccharides [65]. Various strategies have been investigated to reduce the nutritional drawbacks of fiber in canola meal. Dehulling has been shown to improve amino acid digestibility and enhance energy availability in broiler chickens [66]. However, the technique adds costs at the industrial level, limiting its feasibility for large-scale application [67]. An alternative pathway has emerged through plant breeding. Genetic selection has been used to reduce fiber content while increasing protein concentration, providing a longer-term strategy for improving the quality of canola meal [63].

### 3.4. Mineral and Vitamin Level

Canola meal provides not only protein but also appreciable quantities of essential minerals and vitamins, reinforcing its role as a cost-effective alternative to more expensive protein sources. This is particularly relevant in layer hen diets, where its inclusion has consistently supported high egg production [45]. With respect to minerals, canola meal supplies calcium, manganese, iron, selenium, and a variety of trace elements necessary for growth, immune function and reproductive performance in poultry [68,69]. Comparative evaluations suggest that, relative to other oilseed meals of plant origin, canola meal can be considered a richer source of essential minerals [61]. Several studies have specifically reported higher concentrations of calcium and phosphorus compared with soybean meal [70,71]. Although a substantial fraction of phosphorus (approximately 65%) is stored as phytate and therefore poorly available to monogastric animals, the overall contribution of calcium and phosphorus from canola meal remains greater than that provided by soybean meal [70,71]. Additionally, the nutritional limitation of phosphorus stored as phytate is readily addressed through phytase supplementation. The enzyme is frequently included in all commercial poultry diets to release phytate-bound phosphorus, improving both mineral bioavailability and overall nutrient utilization efficiency, while also reducing phosphorus excretion and the potential environmental burden [63,72]. For instance, Kong and Adeola [73] demonstrated, using a regression-based approach, that phytase supplementation enhanced the protein efficiency ratio in broilers receiving canola meal as the sole protein source from days 8 to 15 post hatch. In addition to minerals, canola meal contributes vitamins of nutritional relevance. Canola meal generally contains higher levels of biotin, folic acid, niacin, riboflavin, and thiamin than soybean meal [69]. However, comprehensive datasets describing the full vitamin profile of canola meal remain scarce, and further characterization is needed to accurately define its contribution to poultry diets.

### 3.5. Dietary Inclusion Level

Despite its nutritional advantages, the adoption of canola meal in poultry diets has historically lagged behind soybean meal. This is largely attributed to its lower protein and metabolizable energy content, combined with a higher concentration of dietary fiber [14,56]. High fiber content in canola meal can reduce nutrient utilization and energy digestibility in poultry [41,58]. Fiber-rich diets increase mucin secretion by goblet cells and slow enzyme–substrate interactions, which limits nutrient hydrolysis and absorption [42,43]. Additionally, increased digesta passage associated with high fiber can reduce digestion time and increase endogenous nitrogen losses, further decreasing nutrient availability [13,44]. Advances in plant breeding, particularly the development of cultivars with reduced erucic acid and glucosinolate concentrations, have markedly improved its nutritional value, encouraging greater use in poultry diets [74,75]. Performance trials indicate that dietary inclusion level is a decisive factor. A recent review by Manyeula et al. [76] reported that broilers tolerated canola meal at 1–10% of the diet without adverse effects on feed intake. In contrast, higher inclusion levels (11–40%) were associated with reduced intake [76,77], a response attributed to changes in diet palatability and physical characteristics [78]. Weight gain responses followed a similar pattern. Birds receiving canola meal at inclusion levels up to 40% showed lower growth compared to controls, likely reflecting amino acid imbalances and the impact of fiber on nutrient utilization [22,78,79]. For feed conversion ratio (FCR), Manyeula et al. [76] reported no significant differences when broilers fed diets containing up to 30% canola meal, whereas higher inclusion levels (31–40%) resulted in poorer FCR. In support, Aljuobori et al. [80] found that up to 30% canola meal could be included in broiler diets without impairing growth efficiency, even under chronic heat stress. Collectively, these findings suggest a wide inclusion window of approximately 10–30% canola meal for broiler diets. Further research on processing strategies is needed to enhance the value of canola meal as a soybean substitute and to determine optimal inclusion levels that do not compromise broiler growth performance.

Recommendations for canola meal inclusion in laying hen diets have shifted considerably over time. Early guidelines advised limiting use to approximately 10% [69], a conservative threshold shaped by negative reports on rapeseed meal, including liver damage [81], and observations of numerically higher, though statistically nonsignificant, mortality in hens fed 16.7% canola meal [82]. Also, restrictive recommendations of 11% were proposed for brown-shelled layers due to concerns about a “fishy taint” in eggs [83]. This off-flavor was subsequently traced not to the feed itself, but to a recessive mutation in the flavin-containing monooxygenase-3 (FMO3) gene, which impairs trimethylamine metabolism [83]. Advances in genetic selection have eliminated this defective allele, with ISA confirming in 2010 that all grandparent stock were free of the fishy taint phenotype, and major breeding companies such as Lohmann and Hy-Line later verifying its removal from their brown-egg lines [16]. By 2015, all commercial laying stock derived from these genetic lines in Australia were confirmed to be devoid of the FMO3 mutation, thereby removing a major barrier to higher canola meal inclusions [16]. Under these conditions, Swick [16] demonstrated that cold-pressed canola meal could be safely incorporated at 20% in Hy-Line Brown layer hen diets without adverse effects on egg production or quality. More recent studies have supported these findings, confirming the safety and efficacy of canola meal inclusions up to 20% in layer diets [84,85]. Collectively, these results support the use of canola meal at substantially higher levels than previously recommended, providing a reliable and nutritionally suitable alternative protein source for laying hens without compromising performance or egg quality. However, recent research remains limited, particularly regarding the effects of canola meal from modern cultivars formulated for modern laying hen strains. There is also potential to explore even higher inclusion levels, given the more desirable nutrient profile of canola meal for laying hens compared with broilers.

### 3.6. Anti-Nutritional Factors

The inclusion of canola meal as a plant-based protein source in animal feeds is constrained by the presence of anti-nutritional factors such as phenolic compounds, phytic acid and glucosinolates, which have been linked to reduced growth performance in livestock [23]. Among these, glucosinolates, sinapine, tannins, and phytic acid are considered the primary anti-nutritional constituents of canola meal [64].

Modern canola cultivars have been bred to contain lower glucosinolate levels than traditional rapeseed varieties. Nevertheless, these compounds can still affect feed palatability and nutrient availability, particularly at high inclusion rates [13]. Typical glucosinolate concentrations in canola seeds range from 3.6 to 9.2 μmol/g. Although this is well below levels associated with acute toxicity, high inclusion rates of canola meal in the diet may still negatively impact feed palatability and nutrient availability [11]. Glucosinolate concentrations in canola meal differ considerably across regions. Processing plants in Canada have reported average levels of 3.9 μmol/g on a 10% moisture basis [86], whereas facilities in France reported higher values, averaging 10 μmol/g [87]. A commercial sample from Poland contained approximately 4.3 μmol/g [88]. In Australia, expeller-extracted canola meal was found to contain 7.3 μmol/g on a dry matter basis, with values ranging between 6.3 and 8.1 μmol/g depending on the processing technique [57]. Broiler diets can tolerate inclusion levels of canola meal above the currently recommended level (20%) without adverse glucosinolate-related effects, assuming a conservative maximum of 4 μmol/g feed [13]. Laying hens, however, are more sensitive; dietary glucosinolate levels exceeding 1.5 μmol/g feed may increase the risk of hemorrhagic liver syndrome [89]. Consequently, inclusion of canola meal at 15 to 20% in layer hen diets approaches this safety threshold [13]. However, the introduction of new laying hen strains, together with improved canola cultivars and advances in oil extraction techniques [14,90], presents opportunity to explore inclusion rates of canola meal above 20% in diets for laying hens.

Sinapine, comprising roughly 1% of canola meal, is the main phenolic compound and has been associated with the development of “fishy” off-flavors in eggs [23,81]. This effect is not observed in broiler meat [13]. Further research revealed that the phenomenon is linked not solely to sinapine but to a recessive genetic mutation causing trimethylamine (TMA) accumulation in susceptible hens [83]. Breeding programs have successfully removed this trait from commercial brown-egg layers, substantially reducing the risk of TMA-related taint [85].

Tannins in canola are concentrated primarily in the seed hulls, with darker hull types generally containing higher tannin levels than yellow hulls [91,92]. Previous studies have shown that rapeseed and canola hulls contain between 1.9 and 6.2 g of tannins per 100 g of oil-free material [93]. The dominant fraction consists of insoluble tannins, particularly proanthocyanidins, which are largely responsible for the characteristic dark pigmentation of the seeds [13]. These compounds can interact with dietary proteins and proteolytic enzymes within the gastrointestinal tract, potentially impairing protein digestibility. Nevertheless, because the majority of canola tannins are water-insoluble and remain embedded in the cell wall matrix of the hull fraction, their overall antinutritive impact in animal production is considered to be limited [13].

Phytic acid represents another anti-nutritional factor in canola meal, as phosphorus bound in phytate is poorly digestible for monogastric animals due to low endogenous phytase activity [23]. Supplementing diets with exogenous phytase has been shown to improve phosphorus bioavailability, enhancing overall nutrient utilization and reducing environmental mineral excretion [63,72]. This approach supports both bird production performance and more sustainable feed strategies.

## 4. Strategies for Enhancing the Nutritive Value of Canola Meal

Efforts to improve the nutritional quality of canola meal have focused extensively on plant breeding and processing innovations. Breeding programs aim to develop cultivars with higher protein content and reduced fiber levels. Chen et al. [15] demonstrated that high-protein, low-fiber canola meal exhibited higher CP (49.4% vs. 41.5%) and lower neutral and acid detergent fiber levels than conventional canola meal, resulting in a 5.21% increase in amino acid digestibility. Correspondingly, true metabolizable energy values were higher for the high-protein, low-fiber canola meal (10.27 vs. 9.52 MJ/kg), suggesting selective breeding can enhance feeding value of canola meal. Similar improvements have been reported for yellow-seeded *Brassica napus* varieties, which combine higher oil and protein content with reduced fiber content. Seed color is closely associated with fiber content in canola, with yellow-seeded lines showing higher CP (49.8% vs. 43.8%), lower fiber (24.1% vs. 30.1%), and decreased glucosinolate concentrations (17.1 μmol/g vs. 27.1 μmol/g) relative to black-seeded lines [14,94,95]. These findings indicate that cultivar selection is an effective approach to increase canola meal’s competitiveness as a protein source in poultry diets.

Processing methods play a pivotal role in determining canola meal quality. Solvent extraction can increase CP content but may reduce lysine concentration and amino acid digestibility, particularly during desolventization and toasting steps, where heat exposure is intense [22]. Cold-pressed canola meals generally have higher amino acid digestibility and energy utilization [17], supporting performance outcomes comparable to soybean meal [96]. Extrusion has shown promise in improving digestibility and energy values of canola meal. Ahmed et al. [51] reported an 11.3% increase in amino acid digestibility and a 1.48 MJ/kg gain in energy utilization following extrusion, likely linked to reductions in fiber content in canola meal. Excessive heat, however, remains detrimental; Anderson-Hafermann et al. [49] observed a 43.1% decrease in weight gain and a 32.2% reduction in feed efficiency in broilers fed autoclaved canola meal diets, associated with decreased protein solubility. Preventing heat damage seems to be the most reliable approach for maintaining the nutritional quality of canola meal. Cold-pressed canola meal, which is produced without exposure to high temperatures, typically exhibits better performance in poultry diets and is commonly preferred in Australian poultry production [9].

Enzyme supplementation is widely used in poultry feeding and has shown notable benefits in canola meal-based diets. Phytase alone provides only a slight increase in metabolizable energy, raising it from 14.0 to 14.1 MJ/kg. Xylanase offers a marginally greater effect, bringing it to 14.2 MJ/kg. When both enzymes are combined, the improvement is more pronounced with metabolizable energy reaching 14.7 MJ/kg, and protein digestibility increasing from 75.8% to 78.1% [97]. Interestingly, advances in recombinant DNA technology have produced phytase variants with enhanced functionality, potentially reducing feed costs while improving nutrient utilization [13]. In diets containing 27–29% canola meal, a carbohydrase cocktail dramatically increased non-starch polysaccharide digestibility more than tenfold and led to a 5.47% increase in broiler body weight from day 1 to 20, alongside moderate improvements in feed efficiency [98]. Protease addition also supported broiler growth, with effects more pronounced in canola meal diets than soybean meal diets [99]. In laying hens, protease did not significantly alter performance, egg quality, AMEn, or protein digestibility at inclusion levels of 0, 7.29, or 14.59% canola meal; however, aminopeptidase activity in the duodenum and jejunum increased at 14.59% inclusion [100]. Overall, these findings indicate that enzyme supplementation in canola meal-based diets can enhance nutrient utilization and production outcomes, though effects depend on enzyme type, dosage, and bird species.

Fermentation has emerged as a promising approach to reduce anti-nutritional factors in canola meal and enhance its feeding value. *Lactobacillus* plays a central role in this process. Its activity during fermentation can increase digestive enzyme activity and promote a balanced gut microbiota, both of which support more efficient digestion and nutrient utilization [101]. In many ways, fermented feeds function similarly to probiotics. They help maintain a healthy gut by fostering beneficial microbes, producing organic acids that inhibit harmful species, and preventing pathogenic colonization through competitive exclusion and antagonistic activity [102,103,104,105]. Beyond microbial effects, fermentation induces structural changes in the intestine. Increased villus height and expanded absorptive surfaces improve nutrient uptake and can explain observed gains in growth performance [106]. Fermentation is also one of the most effective strategies to reduce antinutritional factors in unconventional feed ingredients, thereby enhancing their overall nutritional value [104,107]. Several studies have documented increases in CP content, along with reductions in tannins, glucosinolates, and crude fiber following fermentation [108,109,110,111]. Chiang et al. [112] reported that fermentation of rapeseed meal increased broiler body weight gain and feed efficiency. Similarly, Elbaz [106] demonstrated that including fermented canola meal in broiler diets enhanced growth performance, nutrient digestibility, and feed efficiency compared with unfermented meal or canola meal supplemented with probiotics. Fermentation also positively influenced gut health, as indicated by lower *Escherichia coli* counts, higher *Lactobacillus* populations, and improved intestinal morphology in broiler chickens [106]. More recent studies by Elbaz et al. [113] showed that combining fermented canola meal with a multi-enzyme complex including protease, amylase, β-glucanase, xylanase, pectinase, cellulase, and phytase at 0.02% per kg feed further enhanced weight gain and feed efficiency in broiler chickens, likely due to improved nutrient availability, palatability, and immune function. However, outcomes are not universally consistent. Hafeez et al. [114] observed that while 6–12% fermented canola meal increased protein and fiber digestibility, inclusion at 18% negatively affected growth and feed efficiency. Similarly, Xu et al. [115] reported that rapeseed meal fermented with *Lactobacillus fermentum* and *Bacillus subtilis* could safely replace up to 10% of soybean meal in broiler diets. Collectively, these findings indicate that fermentation can enhance the functional and nutritional quality of canola meal, but efficacy depends strongly on inclusion levels, microbial strains, and processing conditions.

In summary, integrated approaches combining selective breeding, processing optimization, enzyme supplementation, and fermentation offer a pathway to improve the nutritional values of canola meal in poultry diets. Cultivars with lower fiber and higher protein content, alongside processing methods that preserve amino acid and energy values, support efficient utilization. Complementary strategies such as enzymatic and microbial interventions further mitigate anti-nutritional effects, highlighting the potential of canola meal as a reliable, competitive protein source in modern poultry production.

## 5. Production Performance, Economic and Environmental Benefits of Canola Meal in Poultry Diets

### 5.1. Production Performance

#### 5.1.1. Utilization of Canola Meal in Standard-Protein Diets

Research evaluating the replacement of soybean meal with canola meal in standard-protein diets for broiler chickens shows variable outcomes, as moderate inclusion has supported improved growth in some studies [78,116], whereas higher levels (generally above 25%) have been linked to reduced nutrient digestibility and performance [117,118,119]. A recent meta-analysis of 15 studies on solvent-extracted canola meal reported that increasing dietary canola meal inclusion led to linear reductions in broiler weight gain and feed efficiency, with predicted declines of about 4% in weight gain and nearly 5% in FCR when soybean meal was replaced with 150 g/kg canola meal. The results also showed substantial variability in bird responses, which likely reflect differences in seed quality, processing conditions, and resultant nutritive value of the meals tested [9]. As mentioned earlier, the intensity and duration of heating during extraction, particularly in desolventization and toasting, can depress amino acid concentrations and digestibility in canola meal, with heat-labile amino acids such as lysine being especially vulnerable [22,49,57]. Although solvent extraction increases CP content, it can also reduce lysine availability and overall amino acid utilization during high-heat stages of processing [22]. In contrast, cold-pressed canola meals tend to retain more digestible amino acids and energy [17]. Seed quality further contributes to nutritional variability; fiber levels show a well-documented negative relationship with energy digestibility [67]. It has been demonstrated that high-protein, low-fiber canola meals contain more CP, has greater amino acid digestibility, and yields higher ME than conventional meal types, indicating that selective breeding can substantially improve feeding value [15]. Collectively, the evidence indicates that while canola meal can be incorporated into broiler diets, both its quality and inclusion rate must be carefully managed to prevent adverse effects on growth performance.

The inclusion of canola meal in layer hen diets has shown considerable promise, with the studies performed reporting positive or neutral impacts on production performance and egg quality. In contrast to broilers, where high crude fiber can restrict nutrient utilization, this characteristic may be advantageous for laying hens as it supports gut health [16]. Importantly, laying hens can tolerate relatively high dietary levels of canola meal provided that total glucosinolate concentrations remain below 1.43 µmol/g [56]. Early research by Leeson et al. [120] demonstrated that complete replacement of soybean meal with canola meal did not compromise laying performance, nutrient retention, or mineral metabolism of laying hens. Recent research has shown that canola meal can replace a significant portion of soybean meal in layer hen diets without compromising production or egg quality [16,84,85]. Cold-pressed canola meal has been included at levels up to 200 g/kg in brown-egg laying hens, with no observed negative effects on egg production, egg weight, feed intake, feed efficiency, or overall egg quality during peak production [16]. At later production stages, inclusion of cold-pressed or expeller canola meal at 150 g/kg slightly reduced feed intake, yet egg mass and laying rate were maintained, resulting in improved feed efficiency compared with solvent-extracted meals [16]. Most measures of egg quality remained unchanged across treatments, although yolk pigmentation was enhanced in hens fed solvent-extracted or expeller meals relative to cold-pressed canola meal [16]. Taken together, these findings indicate that canola meal can be included at levels of up to 20% in layer hen diets without adverse impacts on performance or egg quality, supporting its role as a practical and nutritionally adequate alternative to soybean meal. However, given that practically all laying hen diets in Australia and elsewhere contain canola meal, there are barely any studies performed in modern laying hen strains using current canola cultivars. With the ongoing genetic improvements of both the laying hens and the canola crop, revising the inclusion rates of canola meal for laying hen diets is required.

#### 5.1.2. Utilization of Canola Meal in Reduced-Protein Diets

Interest in reduced-CP diets for broiler chickens has grown considerably in recent years, reflecting a dual emphasis on sustaining productive performance while enhancing the environmental sustainability of poultry production systems [19,20]. Economic factors are also shaping this transition. As the cost of non-bound (crystalline) amino acids continues to decline due to economies of scale, and as soybean meal prices trend upward, the adoption of reduced-CP feeding strategies is expected to become increasingly feasible [21]. Although early research into low protein diets spans several decades, their practical implementation has been constrained by concerns over amino acid imbalances and the risk of performance losses [121]. More recent advances in formulation strategies have addressed many of these issues. The inclusion of crystalline amino acids now allows protein reductions of approximately 2–3 percentage units below breeder recommendations without compromising bird growth or feed efficiency [19,122]. This advancement marks a significant step toward aligning poultry nutrition with sustainability goals, particularly in reducing nitrogen excretion and improving feed efficiency. From an economic standpoint, feed represents the single largest cost in poultry production [123], suggesting that reduced-CP diets could also deliver meaningful cost savings. An additional consequence of lowering CP is the automatic reduction in soybean meal inclusion, a trend with important implications given the sustainability concerns surrounding its production. In this context, partial replacement of soybean meal with canola meal could further strengthen the environmental and economic benefits, offering a combined strategy to meaningfully decrease reliance on soybean meal in poultry diets [9].

Reduced-protein diets that incorporate canola meal offer both economic and environmental advantages. These benefits extend beyond reduced feed costs to include decreases in nitrogen excretion and ammonia emissions, which are key environmental concerns in intensive poultry production [124,125]. When the meal is sourced locally, further gains are achieved by lowering transportation expenses and associated fossil fuel consumption. From a nutritional perspective, canola meal is particularly valuable because of its relatively high concentrations of sulfur-containing amino acids, such as methionine and cysteine, nutrients that are frequently limiting in cereal grains [22,23]. The importance of this characteristic becomes evident in regions like Australia, where wheat dominates poultry feed formulations. Protein reduction in wheat-based diets poses significant challenges due to their amino acid profile [24]. The abundance of sulfur amino acids in canola meal therefore creates an opportunity for nutritional complementarity, particularly when used alongside wheat and other grains in reduced-protein diets. In commercial practice, the total sulfur amino acid (TSAA) requirement in reduced-protein diets is typically satisfied by the inclusion of crystalline methionine, since this is more cost-effective than supplying cysteine [9]. The nutritional assumption underpinning this approach is that methionine can be efficiently converted to cysteine. However, such formulations compress the dietary cysteine-to-methionine ratio [9]. Kalinowski et al. [126] proposed that cysteine should represent about 44% of TSAA in slow-feathering birds and up to 47% in fast-feathering strains. This raises the possibility that reduced-protein diets relying primarily on methionine supplementation may inadvertently depress cysteine to suboptimal levels [9]. Under these circumstances, the naturally higher cysteine content of canola meal offers an important advantage, as it helps to restore a more balanced cysteine-to-methionine ratio, potentially improving the efficacy of reduced-protein feeding strategies. Furthermore, converting methionine to cysteine also creates practical challenges. Because the two amino acids differ in molecular weight, methionine supplies cysteine with only about 80% efficiency on a weight basis. Despite this, many feeding systems still assume a full molar conversion [127,128,129,130,131]. Utilization patterns become even more complex when diets are marginal in methionine. Under such conditions, supplemental methionine and cystine do not contribute equally to meeting the TSAA requirement [132]. For this reason, ingredients that supply appreciable amounts of digestible cystine may help support performance and bodyweight, particularly in laying hens [131]. There is another factor to consider. Methionine is used in several methylation reactions, contributing to the formation of creatine, choline, polyamines, and carnitine [133]. These competing demands reduce the fraction of methionine available for protein accretion. Because cysteine synthesis relies on methionine, providing diets with readily available cysteine sources may spare methionine use. This could increase the amino acid’s availability for body protein growth and other metabolic functions, resulting in a better metabolic efficiency, improved performance, and reduced nitrogen losses [134].

Replacing soybean meal with canola meal as the primary source of intact protein in reduced-protein wheat-based diets necessitates higher canola meal inclusion rates to achieve target CP levels [9]. As a result, wheat and starch contributions to the diet are proportionally reduced. This adjustment carries added benefits, since wheat is characterized by less favorable nutritional traits compared with maize, notably a greater proportion of rapidly digestible starch and higher concentrations of soluble non-starch polysaccharides [24,135]. Reducing wheat inclusions therefore helps to mitigate these limitations. In addition, the partial substitution of rapidly digestible starch with more slowly digestible fractions may improve nutrient utilization and, ultimately, broiler performance [24]. Beyond carbohydrate dynamics, canola meal also contains higher levels of polyunsaturated fatty acids compared with soybean meal, a feature linked to reduced fat deposition in poultry [136]. Such effects are particularly relevant to both broiler and layer hen production, where lean tissue accretion and controlled fat deposition are desirable. However, canola meal is relatively deficient in certain essential amino acids, notably lysine and arginine [13], which necessitates careful diet formulation and targeted supplementation to optimize production performance in birds fed reduced-CP diets.

Over the past several decades, researchers have explored the potential of canola meal to partially or fully replace soybean meal in reduced-protein broiler diets, with particular attention to growth performance, feed efficiency, and product quality. In an early study, Salmon et al. [137] examined the combined effects of canola meal inclusion level, dietary protein level, and nutrient density in wheat-based diets, with added fat used to offset the lower metabolizable energy of canola meal. Their results showed that body weight gain was not influenced by canola meal inclusion or nutrient density. Feed efficiency was also not affected by dietary canola meal level when nutrient density was maintained with fat but declined in low-density formulations. At higher inclusion rates (28.1% in the starter diet and 12.1% in the finisher diet), canola meal reduced sensory quality by lowering chicken flavor intensity and increasing off-flavor incidence, whereas moderate inclusion levels (≤21% in the starter diet and ≤9% in the finisher diet) did not produce adverse sensory effects [137]. Collectively, these findings suggest that canola meal can be incorporated into reduced-protein broiler diets without detriment to growth or feed efficiency, provided that dietary energy is adequately maintained and inclusion rates are carefully controlled to ensure product quality.

The potential of canola meal to replace soybean meal in low-protein broiler diets has also been explored by Ajao et al. [125]. In this study, broilers were fed diets that differed in CP content and protein source during the grower and finisher phases from 10 to 42 days of age. Treatments included a standard corn–soybean meal control diet with adequate CP level (18.5% in the grower and 17.0% in the finisher), a low-protein negative control (14% and 13% CP in the grower and finisher phase, respectively), and two additional diets in which soybean meal was partially or near-completely replaced with canola meal at inclusion levels of 50 or 100 g/kg while maintaining the same reduced CP levels as the negative control. All low-protein diets were formulated to contain similar levels of standardized ileal digestible indispensable amino acids, in accordance with breed-specific recommendations, with glycine and serine included as sources of non-specific nitrogen [125]. Their findings showed that the moderate inclusion of canola meal (50 g/kg) supported growth comparable to the reduced-protein control, whereas higher levels negatively affected performance. Specifically, feed efficiency declined linearly with increasing canola meal inclusion, and carcass weight was depressed in a quadratic manner, while fat deposition decreased linearly. On this basis, the authors concluded that full replacement of soybean meal with canola meal in low-protein diets was not feasible [125]. However, one limitation of the study design was the large reduction in dietary CP level, 4 to 4.5 percentage points lower than the control standard-protein diet, which may have caused deficiencies in non-essential amino acids, altered electrolyte balance, or created insufficient nitrogen supply despite glycine and serine supplementation. These factors could have contributed to the impaired performance, confounding the specific effects of canola meal in the study. In contrast, more recent work by Macelline et al. [138] indicated that canola meal can effectively replace soybean meal in reduced-protein diets containing 19% CP without compromising growth performance, carcass traits, or fat deposition in broiler chickens from 15 to 36 days of age. Moreover, improvements were observed in energy utilization (AME, AMEn), starch digestibility in the distal jejunum, and ileal methionine digestibility [138]. Research on the replacement of soybean meal with canola meal in reduced-protein diets for laying hens remains limited, highlighting the need for further studies to evaluate its effects on production performance, nutrient utilization, and egg quality. Collectively, the available literature evidence suggests that the suitability of canola meal in reduced-protein diets depends heavily on the extent of CP reduction and on careful formulation to ensure adequate amino acid supply. When inclusion levels are optimized, canola meal can serve as a viable and nutritionally advantageous alternative to soybean meal in reduced-protein diets for poultry.

### 5.2. Economic Efficiency and Cost-Effectiveness

#### 5.2.1. Review of Studies on Economic Efficiency and Cost-Effectiveness of Canola Meal Utilization

Local sourcing of feed proteins is increasingly viewed as a practical step toward improving the sustainability of poultry production. Producing feed ingredients closer to farms reduces energy spent on long-distance transport, supports local economies, and lowers risks tied to centralized supply chains [139,140]. Economic benefits are also evident. Reduced transport costs and the use of regionally produced crops or by-products can make local proteins more affordable than imported protein meals, especially as livestock systems work to shrink their environmental footprint [141]. Canola meal provides an example of how local production can align with these goals. In regions where canola processing plants already exist like Australia, its inclusion in poultry diets proves cost-effective compared with soybean meal [16], particularly during periods of high fuel costs or disruptions to global trade. Current market figures place canola meal at about AU$480/ton, equivalent to roughly 70% of the price of soybean meal [142]. Beyond pricing, canola meal contributes to diversification of protein sources. This diversification stabilizes feed supply by reducing dependence on a narrow set of production regions. It also offers protection against shocks related to climate variability, political instability, or transport bottlenecks [143]. Another advantage is predictability. Supply chains built on regional canola production are generally more stable than international markets, which can fluctuate sharply. This stability became especially valuable during events such as the COVID-19 pandemic, when disruptions to global feed systems highlighted systemic vulnerabilities [143]. Looking ahead, the role of canola meal could grow stronger. Advances in enzyme technology and improved feed formulation strategies are expected to increase nutrient availability and further reduce costs. Together, these developments suggest that canola meal can support economic resilience while offering a practical alternative to imported protein sources in poultry diets.

Feed accounts for the largest proportion of production costs in poultry systems, typically representing around 70% of total costs per bird [144,145]. Given the volatility in global soybean meal markets, largely driven by rising demand and constrained supply, there has been growing interest in identifying alternative protein supplements that can maintain productivity while reducing feed costs [78,146]. Canola meal has emerged as a competitive option in this regard, offering both nutritional value and cost efficiency [147]. Harris [147] noted that while canola and soybean meals share comparable nutrient characteristics, their use in practice is often dictated by fluctuations in price and availability. This point is echoed by Swick [16], who highlighted that the relative pricing of canola meal compared to soybean meal determines its economic value in layer hen diets. Earlier work from Agriculture Canada [36] suggested that canola meal could replace up to half of protein supplements in livestock rations without impairing performance, provided market conditions favored its inclusion. These findings underscore the importance of market dynamics in the utilization of canola meal as a sustainable and economical alternative to soybean meal in poultry production.

Recent experimental studies have provided further support for the economic benefits of canola meal in layer hen diets. Swick [16] conducted two experiments evaluating the cost-effectiveness of including cold-pressed, expeller, and solvent-extracted canola meals in wheat-based diets for Hy-Line Brown laying hens. In the first experiment, cold-pressed canola meal was included at 0, 100, and 200 g/kg in diets from 21 to 41 weeks of age, with each treatment comprising 33 replicate cages containing one hen per cage. At the time of the study, the inclusion of 200 g/kg cold-pressed canola meal (priced at $450/mt versus $650/mt for soybean meal [AUD]) reduced feed costs by $25/mt ($AUD), representing a potential annual industry saving of $18 million (AUD) [16]. In a second experiment conducted from 46 to 65 weeks of age, cold-pressed, expeller, and solvent-extracted canola meals were each included at 150 g/kg in the diet, with 32 replicate cages containing one hen per cage per treatment. Feed cost per kg of eggs was lowest in hens fed expeller-pressed canola meal ($0.615 AUD), followed closely by cold-pressed canola meal ($0.619 AUD), and highest in solvent-extracted canola meal ($0.679 AUD) [16]. These findings illustrate that different processing methods impact the economic efficiency of canola meal inclusion, with expeller and cold-pressed meals offering the most favorable cost–benefit ratios.

Economic evaluations of canola meal inclusion in broiler diets have yielded similarly promising outcomes. Klein et al. [148] conducted a cost–benefit analysis indicating that when the price ratio of canola meal to soybean meal falls below 0.7, at least 40 g/kg of canola meal should be incorporated. This recommendation increases to 80 g/kg when the ratio drops below 0.6 [148]. In a more recent study under Egyptian production conditions, Sayed et al. [149] evaluated the economic efficiency of replacing soybean meal with canola meal at varying levels (0–20%) in broiler diets. While higher canola meal inclusion (20%) reduced feed and total production costs, it also led to lower final body weight and feed efficiency, ultimately decreasing revenue. The most economically efficient outcome was achieved with 15% canola meal inclusion, which provided the highest net revenue per bird [149]. Similarly, Abdallah et al. [150] reported that 17% inclusion of canola meal yielded the highest net returns in broiler production. These results highlight the importance of balancing cost savings with performance outcomes, as excessive substitution may diminish profitability despite lower feed costs.

Taken together, the literature evidence indicates that the economic utility of canola meal in poultry production systems is highly context-dependent, shaped by ingredient pricing, processing method, and inclusion rate. When incorporated at moderate levels under favorable market conditions, canola meal can reduce feed costs substantially without undermining bird performance, thereby improving profitability in both broiler and layer hen production.

#### 5.2.2. Cost Analysis of Including Canola Meal in Reduced-Protein Diets via Practical Feed Formulation

**Figure 1:**
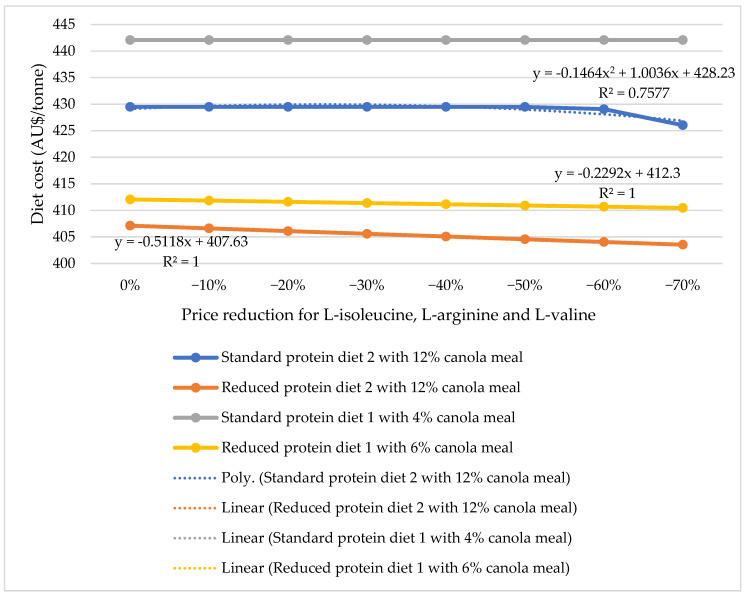
Costs of simulated standard and reduced-protein diets according to stepwise reduced prices of valine, isoleucine and arginine. The grey, orange, and yellow dashed lines are not visible, as they lie exactly on top of the main lines.

Formulating reduced-protein diets generally involves higher inclusions of wheat and supplemental amino acids such as lysine, methionine, and threonine, along with the addition of isoleucine, arginine, and valine. These adjustments are accompanied by reduced use of soybean meal and supplemental oil compared with conventional higher CP formulations [151]. Because soybean meal is partly replaced with crystalline amino acids, the economic feasibility of reduced-protein diets depends heavily on the relative prices of these inputs. Over the past five years, the costs of lysine, methionine, and threonine have remained relatively stable, whereas prices for amino acids such as valine, isoleucine, and arginine have declined due to increasing demand and advances in mass production [21,151]. Therefore, it is pertinent to evaluate how the declining prices of isoleucine, valine and arginine could further enhance the economic efficiency of incorporating canola meal into reduced-protein diets for poultry. To assess this, stimulated standard-protein and reduced-protein diets for Hy-Line Brown laying hens from 60 to 75 weeks of age were formulated, and diet costs were calculated under varying amino acid price scenarios. Specifically, two basal standard-protein diets (2690 kcal AME/kg, 15.8% CP) and two basal reduced-protein diets (2690 kcal AME/kg, 13.8% CP) were developed using current market prices for feed ingredients. All formulations adhered to the nutritional guidelines provided by Hy-Line International [152] and were nutritionally comparable to those used in our previous study, where reduced-protein diets were shown to support optimal laying hen performance (unpublished internal data). The first basal standard-protein diet included 4% canola meal, while the corresponding reduced-protein diet contained 6% canola meal and included only crystalline isoleucine. In this case, the soybean meal level in the reduced-protein diet (7%) was sufficient to meet the birds’ requirements for arginine, valine, and other amino acids while maintaining a least-cost formulation. However, this limited amino acid inclusion may reduce the practical applicability of the reduced-protein diet. To address this, a second set of basal standard-protein and reduced-protein diets was formulated with 12% canola meal. In the reduced-protein formulation, crystalline isoleucine, valine, and arginine were added and the soybean meal inclusion was lowered from 7% to 4%. Subsequently, the prices of isoleucine, valine, and arginine were systematically reduced by 10% increments (i.e., 0%, −10%, −20%, …, −70%) relative to their current market prices. Diet costs were recalculated at each pricing step. At −70% of the current price of valine, isoleucine and arginine, the price of isoleucine was similar to the current price of lysine. The effects of progressively reduced prices of valine, isoleucine and arginine on diet costs in simulated standard-protein and reduced-protein diets are presented in Figure 1.

The detailed ingredient compositions and calculated nutrient profiles of the simulated basal standard-protein and reduced-protein diets are provided in Appendix A Table A1. Results showed that, under current market prices, reduced-protein diets were already less expensive than standard-protein diets. Formulating the reduced-protein diets resulted in higher inclusion of cereal grains (such as wheat, sorghum, and barley) and greater use of crystalline amino acids, including lysine, methionine, threonine, valine, arginine, and isoleucine. At the same time, the levels of soybean meal and added oil were lowered compared with the standard-protein diets. As the prices of valine, isoleucine and arginine were reduced, the cost of reduced-protein diets declined further, though only marginally. This limited cost reduction is attributed to the relatively low inclusion rates of these amino acids in the diets (maximum 0.054%). Diets formulated with 12% canola meal were more cost-effective than those with lower canola meal inclusion. This is due to the interchangeable use of canola meal and soybean meal in the formulations, with canola meal being the less expensive protein source. Interestingly, while the cost of the standard-protein diet containing 4% canola meal remained unaffected by amino acid price reductions, the cost of the standard-protein diet with 12% canola meal decreased when the prices of valine, isoleucine, and arginine were reduced by at least 60%. Specifically, arginine was incorporated into the standard-protein diet with 12% canola meal to reduce soybean meal inclusion when its price dropped by at least 60%. Therefore, the reducing cost of valine, isoleucine, and arginine would help to reduce the cost of reduced-protein diets slightly in the future, but this is limited due to the small inclusion rates. However, when the amount of canola meal in the diet increased, more amino acids were incorporated, reducing the diet cost. These findings underscore the importance of both ingredient selection and amino acid market dynamics in optimizing the cost-effectiveness of reduced-protein poultry diets, particularly when higher levels of canola meal are utilized.

### 5.3. Environmental Benefits

#### 5.3.1. Greenhouse Gas Emissions and Carbon Footprint

Poultry production is often regarded as a relatively sustainable livestock sector due to its comparatively low environmental footprint [153,154]. Nevertheless, the industry faces notable environmental pressures. Ammonia emissions from housing and manure management contribute to acidification, while nitrate leaching from land-applied manure exacerbates eutrophication [155,156,157]. Feed composition and sourcing are key determinants of overall greenhouse gas (GHG) emissions in poultry systems [158]. Soybean meal remains the dominant protein source, yet concerns regarding its economic, environmental, and social sustainability are growing, particularly in arid and semi-arid regions [77,159]. In many developing countries, regulatory and logistical barriers further limit access, especially delays associated with GMO import approvals. Given that most soybean meal is sourced from the United States and Brazil where GMO varieties prevail, shipments are sometimes held at ports for extended periods [160]. These constraints underscore the need for alternative protein sources that are locally accessible, cost-efficient, and nutritionally viable. Canola meal represents one such option. In addition, climate change compounds these challenges. Projections indicate that US soybean yields could decline by 86–92% by 2050 under certain climate scenarios [161]. This expected reduction reinforces the importance of diversifying protein sources, with canola meal offering a more climate-resilient option in regions where agro-climatic conditions favor its cultivation [162].

Greenhouse gas emissions are typically quantified using the Global Warming Potential (GWP), which incorporates the contributions of carbon dioxide (CO_2_), nitrous oxide (N_2_O), and methane (CH_4_) over a 100-year time frame [163]. In this system, 1 kg of CH_4_ and N_2_O are equivalent to 25 and 298 kg of CO_2_, respectively, and the cumulative GWP per functional unit is often referred to as the “carbon footprint.” Incorporating alternative protein sources, such as peas, beans, or rapeseed, into broiler diets has been projected to reduce GWP by as much as 12% at inclusion rates of 10–30% [164]. Beyond serving as a feed component, rapeseed/canola also offers agronomic benefits when used in rotation. It can lower nitrogen fertilizer requirements, suppress weeds and pests, and enhance yields in subsequent crops [165,166,167]. Expanding domestic production of rapeseed/canola could therefore simultaneously improve land-use efficiency and reduce overall GHG emissions in animal production systems [158]. Producing protein meal locally, for instance canola meal, avoids nearly 62% of emissions associated with long-distance transport of imported soybean meal [158]. This benefit becomes even more pronounced when feed ingredients traverse extensive international supply chains. Frequent rotation with break crops such as peas and rapeseed can also reduce diesel use on farms, since lighter stubble loads and fewer disease residues allow for reduced tillage operations [158,165]. However, there are trade-offs. Diets containing higher proportions of wheat bran, oats, or rapeseed meal may reduce nutrient digestibility. Lower digestibility can increase manure output, which in turn increases methane emissions during manure management [158]. Therefore, while alternative protein sources offer clear environmental advantages, careful formulation and management are essential to avoid unintended consequences.

#### 5.3.2. Resource Efficiency and Biodiversity Implications

From a sustainability perspective, canola meal offers several notable advantages as an alternative protein source in poultry diets. Its inclusion can improve the economic efficiency of intensive production systems while simultaneously lowering input requirements, as canola cultivation generally requires less irrigation, fertilizer, and pesticide application compared with soybean production [168]. These traits render it particularly suitable for semi-arid regions characterized by low soil fertility. In European systems, substituting imported soybean meal from South America with locally produced protein sources has been identified as a key approach to reduce the environmental footprint of monogastric livestock operations [169]. Furthermore, studies have shown that integrating regional feedstuffs, such as canola meal, alongside synthetic amino acid supplementation can markedly decrease the carbon footprint of animal diets [170]. Canola meal also exemplifies resource-efficient co-production, deriving both oil and protein from the same crop. This dual output maximizes resource utilization while minimizing waste, consistent with circular economy principles [171,172]. By partially replacing crops grown solely for feed, canola meal reduces competition with human food production and supports more sustainable agricultural practices.

Rapeseed (canola) cultivation plays a key role in supporting agroecological diversity by providing floral resources that sustain pollinator populations [173]. Beyond serving as a major oilseed crop, rapeseed offers abundant nectar, a resource valued by bees and other pollinators. Its relatively long flowering period ensures prolonged nectar availability, facilitating both production and collection. The nectar itself contains high concentrations of easily utilizable sugars, contributing to its nutritional attractiveness. Additionally, the characteristic floral aroma, associated with compounds such as 3-phenylpropionic acid and phenylacetic acid, further enhances pollinator visitation [174]. When managed sustainably, canola fields not only function as effective pollinator habitats but also support broader agroecosystem health. Such practices improve crop rotation outcomes, enhance soil structure, and promote nutrient cycling, creating synergistic benefits across the agricultural landscape [167].

#### 5.3.3. Other Environmental Benefits

Rapeseed (canola) cultivation offers notable environmental benefits, particularly through its potential for phytoremediation of soils contaminated with heavy metals. Globally, heavy metal pollution represents a major challenge. In Western Europe, approximately 1.4 million sites are reported as contaminated [175], while in China, around 45 million hectares (roughly one-sixth of arable land) are affected by heavy metals [176]. Among the various phytoremediation options, rapeseed has emerged as a highly effective candidate for cadmium (Cd) remediation due to several key attributes [173]. It demonstrates a strong capacity for heavy metal uptake, with Cd accumulation in vegetative tissues reaching levels up to 2000 mg/kg, while maintaining low levels in seeds [177,178]. High biomass yield, ease of harvest, and broad climatic adaptability further enhance its suitability for large-scale applications [173]. In addition to its phytoremediation potential, rapeseed oil provides economic value, increasing the feasibility of integrating this crop into farming systems. Breeding programs targeting cultivars that sequester high Cd levels in vegetative tissues while maintaining low Cd concentrations in seeds offer dual benefits: effective soil decontamination and potential financial returns for farmers [173]. The crop’s relatively simple agronomic requirements also support Cd uptake, making it an accessible and cost-effective option. Collectively, these factors underscore rapeseed as an environmentally sustainable and practical strategy for addressing Cd-contaminated soils, with significant potential for broader adoption.

Despite these benefits, canola cultivation is not without trade-offs. Expanding production domestically may reduce land-use pressures in biodiversity-sensitive regions, yet it can simultaneously increase total land requirements. In certain systems, it may also contribute more strongly to marine eutrophication than soybean cultivation [179]. Furthermore, the environmental impact of canola production is strongly influenced by field management and local agronomic practices, and this may differ from patterns seen in soybean systems. These considerations highlight the need for region-specific life cycle assessments and the implementation of sustainable management strategies to ensure environmentally responsible expansion of canola production. Further work is also needed to refine life cycle assessment approaches specific to canola meal used in poultry diets. Improved methods would help quantify potential environmental advantages and clarify where efficiencies can be gained. These evaluations should account for differences in cropping systems, transport distances, and final use so that comparisons with other protein ingredients are based on consistent and realistic assumptions.

## 6. Future Directions and Research Opportunities

The lack of data pertaining to laying hens despite the ubiquitous use of canola meal in laying hen diets is staggering. Advancing feeding strategies that incorporate canola meal presents an important opportunity to improve efficiency, sustainability, and resilience in poultry production. To achieve this, future investigations should prioritize research for laying hens, and in particular, optimization of amino acid ratios tailored to bird type and production stage, alongside the evaluation of feed additives that enhance digestibility and nutrient utilization. Long-term performance studies, supported by economic modeling and standardized methods for quantifying environmental impacts, will be essential to confirm the viability of these approaches under commercial conditions.

Achieving consistent outcomes with reduced-protein canola meal-based diets will require precise formulation coupled with close monitoring of bird performance. In addition to growth and efficiency metrics, attention should be given to ingredient costs, supplementation strategies, and potential improvements in nutrient conversion. Such data will allow for progressive refinement of diet formulations and optimization of broader feeding programs. Parallel to these efforts, life cycle assessment frameworks specific to canola-based poultry diets should be developed to assess environmental benefits across different production contexts.

The application of precision nutrition tools, including sensor-based monitoring and adaptive formulation software, offers considerable promise for improving nutrient delivery accuracy. These technologies enable rapid adjustments to dietary inputs in response to bird performance or environmental variation, thereby supporting more efficient use of feed resources. At the same time, exploring interactions between canola meal and other functional feed additives such as enzymes, probiotics, or prebiotics may uncover complementary effects on gut health, immunity, and nutrient absorption, ultimately improving both bird productivity and welfare.

Recent interest in the gut microbiome further underscores the potential of canola meal in reduced-protein diets. Its content of fermentable fiber and bioactive compounds suggests possible prebiotic activity, which could shape microbial composition and function. Since microbial populations influence energy extraction, amino acid metabolism, and immune responses, clarifying the role of canola meal in microbiome development may provide new insights into its contribution to bird performance.

From an economic perspective, the feasibility of canola meal inclusion depends on shifting market dynamics. Factors such as the cost and availability of alternative protein sources, regulatory requirements, and consumer-driven trends will influence its competitiveness. Economic models that incorporate sensitivity analyses across diverse market scenarios will be important for identifying risks and guiding cost-effective use. In addition, value-added innovations such as production of protein concentrates, enzymatically modified meals, or specialized functional ingredients could further enhance the economic appeal of canola meal. Locally integrated supply chains may also provide long-term stability by reducing dependence on imported raw materials and improving regional profitability.

## 7. Conclusions

This review synthesizes current knowledge on canola meal use in poultry nutrition and clarifies its nutritional, economic, and environmental implications across poultry species. Available research demonstrates that canola meal is a viable and adaptable alternative protein source in poultry diets, providing nutritional, economic, and environmental benefits when applied appropriately. Its amino acid profile is well-balanced, supporting growth and production. However, higher fiber content and anti-nutritional compounds reduce metabolizable energy, making it more suitable as a protein source for laying hen diets than for broiler diets. Advances in processing, enzyme supplementation, fermentation, and modern cultivars have improved its nutritional quality and practical utility. Responses to canola meal inclusion in poultry diets vary by species. Broilers show inconsistent growth performance at higher inclusion levels, whereas laying hens tolerate up to 20% without negative effects on egg production or egg quality. Economically, canola meal is cost-competitive with soybean meal. From an environmental perspective, replacing imported soybean meal with local canola reduces greenhouse gas emissions, improves resource efficiency, and supports pollinator populations. Trade-offs remain, including increased land use, variable digestibility, and potential contributions to eutrophication; thus, careful management is required to maximize benefits. Incorporating canola meal into reduced-protein diets offers both economic and ecological benefits, though effectiveness depends on the extent of CP reduction and the precision of amino acid formulation. Overall, canola meal strengthens the sustainability and resilience of poultry production systems when inclusion levels are matched to species requirements and regional conditions. Opportunities appear especially promising in laying hens, owing to the more favorable nutritional characteristics of canola meal for hens compared with broilers and the capacity of laying hens to tolerate higher inclusion levels. Despite this promise, scientific evidence specific to laying hens, particularly in the context of reduced-protein diets, remains scarce. Targeted research in this area is needed and could yield substantial economic and environmental benefits for egg production systems. Future studies should determine the maximum and optimal inclusion levels of canola meal from modern cultivars in both standard- and reduced-protein diets formulated for modern laying hen strains. This includes evaluating its use under dietary interventions such as fermentation, enzyme supplementation, and/or probiotic supplementation. Such research should assess effects on birds’ performance, product quality, and health conditions to fully characterize the value of canola meal in laying hen nutrition.

## Figures and Tables

**Table 1:** Nutrient profiles of canola meal and soybean meal (as-fed basis) ^1^.

Nutrient Content (%)	Canola Meal (Australia)			Soybean Meal (USA)
	Cold-Pressed	Expeller	Solvent Extract	
Dry matter	92.25	91.82	90.81	88.68
Crude protein	36.59	35.30	36.58	42.34
Crude fat	12.53	9.37	2.87	1.94
Crude fiber	10.44	12.63	11.80	4.69
Lysine ^2^	2.09	1.92	2.00	2.68
Methionine	0.61	0.63	0.65	0.66
Threonine	1.49	1.55	1.62	1.86
Cysteine	0.88	0.82	0.82	0.70
Tryptophan	0.46	0.44	0.48	0.88
Arginine	2.04	1.94	2.19	3.50
Valine	1.78	1.80	1.90	2.16
Isoleucine	1.37	1.40	1.51	2.20
Leucine	2.31	2.39	2.54	3.65
Histidine	0.98	0.99	1.05	1.24

^1^ Adapted from Moss [46]; ^2^ Values of amino acids are total amino acid content.

## Data Availability

The original contributions presented in this study are included in the article. Further inquiries can be directed to the corresponding author.

## Appendix A

**Table A1:** Diet composition and calculated nutrient values of simulated basal standard and reduced-protein diets used for diet cost estimates (as-is basis).

Ingredients, %	Standard-Protein Diet 1 with 4% Canola Meal	Reduced-Protein Diet 1 with 6% Canola Meal	Standard-Protein Diet 2 with 12% Canola Meal	Reduced-Protein Diet 2 with 12% Canola Meal
Wheat	30.0	36.0	30.0	33.0
Soybean meal	15.0	7.0	10.0	4.0
Sorghum	28.0	30.0	27.0	28.0
Canola meal	4.00	6.00	12.0	12.0
Barley	12.0	9.00	10.0	12.0
Limestone grit	6.80	6.80	6.80	6.80
Fine limestone	3.17	3.20	2.90	2.97
Canola oil	0.89	0.00	0.62	0.00
Salt	0.152	0.087	0.138	0.068
Sodium bicarbonate	0.100	0.200	0.100	0.212
Monocalcium phosphate	0.100	0.134	0.100	0.114
Choline chloride 60%	0.118	0.146	0.137	0.160
L-lysine HCl	0.022	0.216	0.065	0.249
D,L-methionine	0.139	0.164	0.103	0.145
L-threonine	0.000	0.054	0.000	0.057
L-isoleucine	0.000	0.033	0.000	0.054
L-arginine	0.000	0.000	0.000	0.017
L-valine	0.000	0.000	0.000	0.003
Layer vitamin-mineral premix ^1^	0.100	0.100	0.100	0.100
Enzyme Axtra XB 201 TPT	0.010	0.010	0.010	0.010
Enzyme Axtra PHY Gold 10,000 FTU/g	0.006	0.006	0.006	0.006
Pigment Jabiru red	0.004	0.004	0.004	0.004
Pigment Jabiru yellow	0.003	0.003	0.003	0.003
Total	100	100	100	100
Formula cost (AU$/tonne)	442	412	430	407
Calculated nutrient content, % (otherwise as indicated)				
Dry matter	90.2	89.9	90.1	89.9
AME_n_ ^2^, kcal/kg	2690	2690	2690	2690
Crude protein	15.8	13.8	15.8	13.8
Crude fat	2.67	1.92	2.58	2.02
Crude fiber	3.06	3.14	3.84	3.84
Ash	9.41	9.15	9.52	9.27
Digestible ^3^ lysine	0.670	0.670	0.670	0.670
Digestible methionine	0.369	0.374	0.347	0.363
Digestible methionine + cysteine	0.600	0.600	0.600	0.600
Digestible threonine	0.496	0.470	0.493	0.470
Digestible isoleucine	0.605	0.530	0.578	0.530
Digestible leucine	1.177	1.015	1.139	0.979
Digestible tryptophan	0.184	0.156	0.182	0.154
Digestible arginine	0.878	0.702	0.843	0.690
Digestible histidine	0.333	0.284	0.334	0.274
Digestible valine	0.692	0.593	0.686	0.590
Digestible glycine	0.517	0.446	0.532	0.439
Digestible phenylalanine	0.655	0.545	0.626	0.503
Calcium	4.061	4.061	4.000	4.000
Available phosphorus	0.337	0.320	0.342	0.320
Sodium	0.160	0.160	0.160	0.160
Chloride	0.173	0.173	0.170	0.170
Choline, mg/kg	1600	1600	1600	1600
Linoleic acid	1.20	1.03	1.27	1.12

^1^ Vitamin-mineral premix provided the following per kilogram diet: vitamin A, 10,000 IU; vitamin D, 3000 IU; vitamin E, 20 mg; vitamin K, 3 mg; nicotinic acid (niacin), 35 mg; pantothenic acid, 12 mg; folic acid, 1 mg; riboflavin (B2), 6 mg; cyanocobalamin (B12), 0.02 mg; biotin, 0.1 mg; pyridoxine (B6), 5 mg; thiamine (B1), 2 mg; copper, 8 mg as copper sulphate pentahydrate; cobalt, 0.2 mg as cobalt sulphate 21%; molybdenum, 0.5 mg as sodium molybdate; iodine, 1 mg as potassium iodide 68%; selenium, 0.3 mg as selenium 2%; iron, 60 mg as iron sulphate 30%; zinc, 60 mg as zinc sulphate 35%; manganese, 90 mg as manganous oxide 60%; antioxidant, 20 mg; ^2^ AMEn: N corrected apparent metabolizable energy; ^3^ Digestible amino acid coefficients of conventional feed ingredients were determined by Near-Infra Red spectroscopy (Foss NIR 6500, Denmark) standardized with Adisseo calibration.
